# Altered neural flexibility in children with attention-deficit/hyperactivity disorder

**DOI:** 10.1038/s41380-022-01706-4

**Published:** 2022-07-22

**Authors:** Weiyan Yin, Tengfei Li, Peter J. Mucha, Jessica R. Cohen, Hongtu Zhu, Ziliang Zhu, Weili Lin

**Affiliations:** 1 Biomedical Research Imaging Center, University of North Carolina at Chapel Hill, Chapel Hill, NC USA; 2 Department of Radiology, University of North Carolina at Chapel Hill, Chapel Hill, NC USA; 3 Department of Mathematics, Dartmouth College, Hanover, NH USA; 4 Department of Mathematics, University of North Carolina at Chapel Hill, Chapel Hill, NC USA; 5 Department of Psychology and Neuroscience, University of North Carolina at Chapel Hill, Chapel Hill, NC USA; 6 Carolina Institute for Developmental Disabilities, University of North Carolina at Chapel Hill, Chapel Hill, NC USA; 7 Department of Biostatistics, University of North Carolina at Chapel Hill, Chapel Hill, NC USA

**Keywords:** ADHD, Diagnostic markers

## Abstract

Attention-deficit/hyperactivity disorder (ADHD) is one of the most common neurodevelopmental disorders of childhood, and is often characterized by altered executive functioning. Executive function has been found to be supported by flexibility in dynamic brain reconfiguration. Thus, we applied multilayer community detection to resting-state fMRI data in 180 children with ADHD and 180 typically developing children (TDC) to identify alterations in dynamic brain reconfiguration in children with ADHD. We specifically evaluated MR derived neural flexibility, which is thought to underlie cognitive flexibility, or the ability to selectively switch between mental processes. Significantly decreased neural flexibility was observed in the ADHD group at both the whole brain (raw *p* = 0.0005) and sub-network levels (*p* < 0.05, FDR corrected), particularly for the default mode network, attention-related networks, executive function-related networks, and primary networks. Furthermore, the subjects with ADHD who received medication exhibited significantly increased neural flexibility (*p* = 0.025, FDR corrected) when compared to subjects with ADHD who were medication naïve, and their neural flexibility was not statistically different from the TDC group (*p* = 0.74, FDR corrected). Finally, regional neural flexibility was capable of differentiating ADHD from TDC (Accuracy: 77% for tenfold cross-validation, 74.46% for independent test) and of predicting ADHD severity using clinical measures of symptom severity (*R*^2^: 0.2794 for tenfold cross-validation, 0.156 for independent test). In conclusion, the present study found that neural flexibility is altered in children with ADHD and demonstrated the potential clinical utility of neural flexibility to identify children with ADHD, as well as to monitor treatment responses and disease severity.

## Introduction

Attention-deficit/hyperactivity disorder (ADHD) is one of the most prevalent psychiatric disorders in children, affecting 3–5% of children worldwide [1, 2]. ADHD is characterized by developmentally inappropriate symptoms of inattention, impulsivity, and hyperactivity. Children with ADHD exhibit difficulties controlling their behaviors and attention, which affects their academic performance and social functioning [3]. Importantly, these symptoms often persist into adulthood [4]. Current ADHD diagnosis relies largely on behavioral assessments after symptoms onset. In addition, clinical diagnostic approaches could be subjective and thus may influence diagnostic accuracy. Thus, approaches capable of early and objective diagnosis of ADHD may provide an opportunity of early intervention to potentially minimize its long-term sequelae [5–7]. The development of machine learning methods has provided the opportunity to solve these concerns. By integrating neuroimaging data and machine learning methods, it is possible to differentiate subjects with ADHD from typically developing children (TDC) and, critically, to predict clinical outcomes [8].

A large number of neuroimaging studies have observed that symptoms of ADHD are driven by atypical brain network organization and impaired functional connectivity (FC) [9–12]. Disrupted whole brain and sub-network FC [9, 13], altered small-world topology, higher local and lower global efficiency [14], and, finally, reduced segregation between default mode network (DMN) and task-relevant networks [15, 16] have been reported in ADHD. However, one of the common assumptions of the aforementioned studies was that the brain is temporally stable during the entire imaging acquisition period (5–8 min). Recent evidence indicates that subjects are likely to engage in several types of mental activities during a resting period of imaging acquisition [17, 18], which could result in altered functional brain network organization throughout the course of the scan [19, 20]. More importantly, several lines of evidence have reported that brain dynamics are relevant for complex cognitive processes [21–23] and are related to psychiatric and neurologic disease [24, 25]. Thus, measuring “dynamic” brain features through estimation of time-related variations across multiple short time windows has gained substantial interest [24]. Notably, altered dynamic brain states, distorted quasi-periodic patterns of brain activity, and changes in temporal variability of FC have been observed in ADHD [26–30].

Cognitive flexibility is a critical aspect of human cognition [31] and has been reported to be a biomarker for brain disorders [32–35]. Children with ADHD have reduced cognitive flexibility as compared to TDC (higher switch costs and slower reaction time) [36–38]. Recent studies have suggested that brain dynamics are important features underlying cognitive flexibility [21, 23, 39–41]. Specifically, neural flexibility, calculated as the frequency at which brain regions change their allegiance from one functional module to another during fMRI acquisition, has recently been proposed [42, 43]. Neural flexibility not only potentially links to cognitive flexibility, but also has been reported to predict learning outcomes and executive functions in healthy subjects [42, 44]. Therefore, neural flexibility might be a useful metric to reflect impaired cognitive flexibility in ADHD subjects.

In this study, we aimed to determine whether neural flexibility can serve as a biomarker to differentiate children with ADHD from TDC and whether it is associated with ADHD severity. Specifically, we implemented machine learning methods on neural flexibility estimates to: (1) distinguish children with ADHD from TDC; and (2) to assess symptom severity in children with ADHD. We hypothesized that children with ADHD would exhibit lower neural flexibility than that of TDC and that it would successfully differentiate groups and predict symptom severity. Furthermore, although pharmacological treatments can ameliorate the core symptoms of ADHD and improve subjects’ future functional outcomes [45], only a few studies have investigated how medication use impacts functional network organization in ADHD [46]. Therefore, we additionally hypothesized that children with ADHD who were on medication would show a “recovery” of neural flexibility toward that observed in TDC.

## Methods

We used two sites from a publicly available, multi-site ADHD dataset, the ADHD-200 study [47]: Peking University (PKU) and New York University (NYU), with 236 and 192 subjects, respectively. The experimental protocols were approved by the local Internal Review Board. Written informed consent was obtained from all participants. The PKU and NYU study cohorts are the two largest samples and have balanced numbers of ADHD and TDC subjects. The ADHD Rating Scale IV [48] and Conner’s Parent Rating Scale-Revised, Long version (CPRS-LV) [49] were used by PKU and NYU respectively to clinically assess the severity of ADHD. Exclusion criteria included left-handedness, IQ below 80, no ADHD rating scale, loss of consciousness due to head trauma, neurological illness, schizophrenia, affective disorder, pervasive development disorder, or substance abuse. In addition, subjects who failed to pass quality control of image preprocessing were also excluded from the analysis, including no full brain coverage, failed tissue segmentation, failed image registration, and excess motion (mean FD > 0.3 mm, maximal head motion of more than 5 mm or 5 degrees). Children with ADHD were included whether or not they were currently taking stimulant medication. In main analyses, all children with ADHD were included in a single group. We additionally investigated the effect of stimulant medication use on neural flexibility by separating children with ADHD into medicated and unmedicated groups. For medicated group, psychostimulant medications were withheld 24–48 h prior to scanning.

RsfMRI data were preprocessed using FSL [50], which included discarding the first 10 volumes, slice-timing correction, motion correction with the mcflirt function of FSL [50], spatial smoothing (6 mm full-width at half-maximum), bandpass filtering (0.01 Hz–0.08 Hz), global mean/white matter/cerebrospinal fluid (CSF) signal regression, 24 head motion parameters regression and wavelet denoising [51, 52]. The time series lengths varied among subjects and imaging sites. To minimize biases contributed by the varying lenghts of time series data, the total time series length was kept at 225 for PKU subjects and 165 for NYU subjects. For each subject, T1-weighted images were first segmented into three tissue types, including gray and white matter and CSF. The tissue segmentation images were then normalized to a standard template using the advanced normalization tools (ANTs) [53].

After preprocessing, a 5 mm sphere around the coordinates defined by Power264 atlas [54], was deformed back to the rsfMRI space to extract the mean time series of each region of interest (ROI). Specifically, Power264 atlas parcellates the brain into 264 regions and 14 functional systems, including sensorimotor hand (SH), sensorimotor mouth (SM), auditory (AUD), visual (VIS), cingulo-opercular (CO), frontoparietal (FP), default model (DMN), memory retrieval (MEM), salience (SAL), subcortical (SUB), ventral attention (VA), dorsal attention (DA), cerebellar (CB), as well as an uncertain system (UC). A sliding window approach with a window width of 30 volumes and an increment of 1 volume was employed for the time series data. Pearson’s correlations were calculated for each pair of the 264 ROIs in each window, a *p* value for each correlation coefficient was estimated using the MATLAB function corrcoef, and only connections significantly different from zero were retained (*p* < 0.05, FDR corrected for all 34,716 connections).

**Fig. 1:**
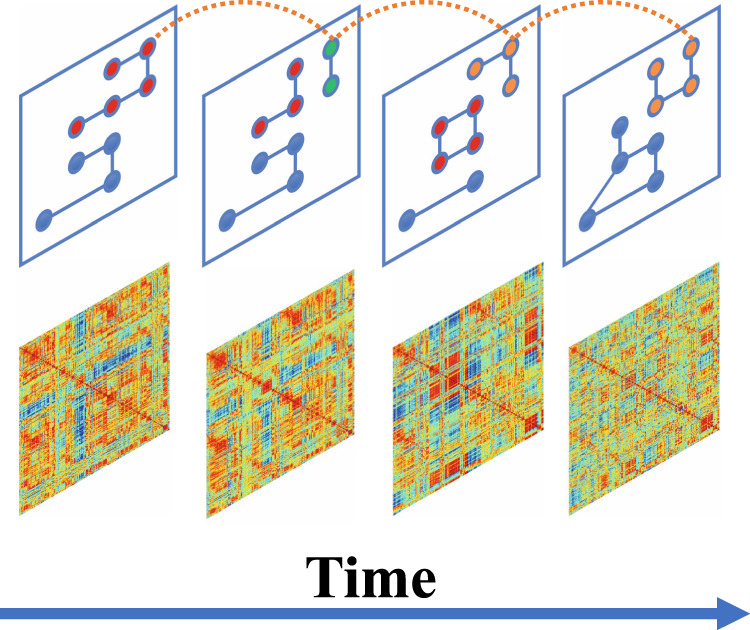
Illustration of a multilayer network. Top panel: in the multilayer network representation of temporal data, each node is connected to itself in adjacent contiguous windows. Next, each node is assigned to a functional community, represented by different colors. Bottom panel: representative correlation matrices of each sliding window.

A multilayer network was constructed by connecting each node to itself in adjacent time windows (Fig. 1). Dynamic community detection based on multilayer modularity was performed on the weighted multilayer network using the Generalized Louvain method [55, 56]. The Generalized Louvain algorithm outputs a community assignment for each node in each time window. Using the community assignment results, neural flexibility was calculated [42, 57] as:

$$f_i = \frac{{n_i}}{N}$$

where *N* is the total number of possible community changes and *n*_*i*_ is the number of times node *i* changes its community label. To account for pseudo-randomness of the community detection algorithm, the Generalized Louvain method was repeated 100 times and mean values of all community-based measures were taken. Whole brain and network-level neural flexibility were calculated as the mean neural flexibility of all nodes of the entire brain and within a predefined canonical network from the Power264 atlas, respectively.

Linear regression was applied to evaluate statistical differences between groups (e.g., ADHD vs TDC, unmedicated ADHD vs medicated ADHD, unmedicated ADHD vs TDC, medicated ADHD vs TDC). Age, sex, mean FD, and imaging site were included as covariates. Statistical significance was considered as *p* < 0.05. False discovery rate (FDR) correction was performed for multiple comparisons. The extreme gradient boosting (XGBoost) algorithm was used to develop two models: a classification model to differentiate subjects with ADHD from TDC, and a regression model to predict ADHD severity of individuals. Optimally predictive combinations of region-wise neural flexibility were identified based on their ranked nodal importance for the classification and regression models, respectively. Specifically, we evaluated the model performance of the top N (${{{{{{{\mathrm{N}}}}}}}} \in [1,264]$) regions based on their importance scores using tenfold cross-validation (CV), which we conducted ten times. Through this process a set of brain regions yielding the best performance of accuracy for the classification model and *R*^2^ for the regression model was determined. Tuning parameters of the final models were provided in Table S2.

Additional details and analyses are provided in Supplementary Information.

## Results

After data preprocessing and quality control, a total of 180 ADHD subjects and 180 TDC were included in the statistical analyses. Demographic and clinical information, as well as motion parameter estimates, are summarized in Table 1.

**Table 1:** Demographic, clinical and motion information for TDC and ADHD subjects.

	PKU			NYU				*P* Value	
	TDC	ADHD		TDC	ADHD			TDC vs ADHD	PKU vs NYU
*Medication Status*	Naïve	Naïve	Non-Naïve	Naïve	Naïve	Unknown	Non-Naïve		
*N* *(M/F)*	111 (65/46)	58 (51/7)	25 (23/2)	69 (31/38)	25 (16/9)	51 (38/13)	21 (17/4)	*P* < 0.001	0.04
*Age, mean (STD), years*	11.27 (1.80)	12.29 (2.07)	11.68 (2.08)	11.24 (3.16)	10.48 (2.39)	10.92 (2.30)	13.07 (2.98)	0.95	0.88
*Age range, years*	8–15	8–17		7–18	7–18				
*IQ Measurement*	Wechsler Intelligence Scale for Chinese Children-Revised			Wechsler Abbreviated Scale of Intelligence					
*IQ, mean (STD)*	117.72 (13.48)	104.06 (11.19)	110 (14.58)	110.30 (13.97)	107.04 (13.88)	107.44 (15.60)	101.43 (12.29)	*P* < 0.001	*P* = 0.0016
*ADHD Measurement*	ADHD Rating Scale IV			Conners’ Parent Rating Scale-Revised, Long version					
*ADHD Index, mean (STD)*	29.78 (6.46)	48.40 (7.2)	52.28 (9.45)	45.26 (6.48)	72.12 (9.36)	72 (9.37)	68.80 (7.47)	*P* < 0.001	*P* < 0.001
*FD, mean (STD), mm*	0.11 (0.04)	0.13 (0.05)	0.13 (0.06)	0.11 (0.05)	0.14 (0.07)	0.13 (0.04)	0.12 (0.06)	*P* < 0.001	0.7

### Neural flexibility

**Fig. 2:**
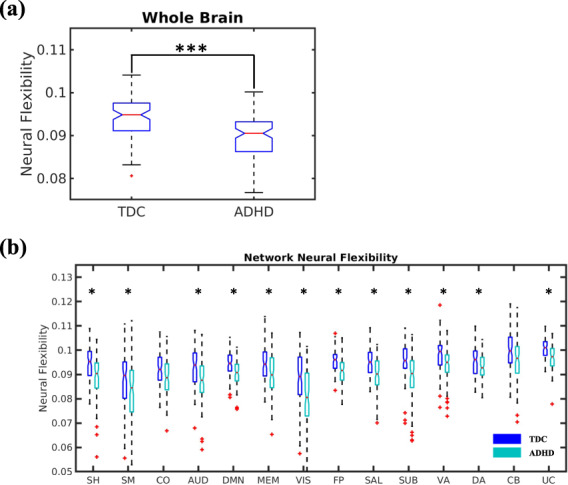
Alteration of neural flexibility in ADHD. **a** A boxplot shows significantly decreased whole brain neural flexibility in subjects with ADHD as compared to TDC. **b** Comparisons of neural flexibility of different functional networks. Black asterisks indicate significant differences after FDR correction. SH sensorimotor hand, SM sensorimotor mouth, CO cingulo-opercular, AUD auditory, DMN default mode, MEM memory retrieval, VIS visual, FP frontoparietal, SAL salience, SUB subcortical, VA ventral attention, DA dorsal attention, CB cerebellar, UC uncertain system. Statistical significance levels: *p < 0.05, **p < 0.01, ***p < 0.001.

Significantly decreased whole brain neural flexibility was observed in subjects with ADHD as compared to TDC (raw $p = 0.0005$; Fig. 2a). Consistent with this finding, we observed that modules were significantly more stable in subjects with ADHD (Fig. S1). To further examine if the observed decrease in neural flexibility in ADHD was driven by specific functional brain systems or a was general feature of the whole brain, network-level neural flexibility was compared between ADHD and TDC subjects (Fig. 2b). Compared to TDC, subjects with ADHD exhibited significantly decreased neural flexibility in all but the CO and CB networks (*p* values < 0.05, FDR corrected for 14 networks). Additional region-level analysis revealed similar patterns (Fig. S2). Together, these results indicate that subjects with ADHD exhibited reduced neural flexibility spanning across multiple functional networks encompassing both higher order and basic cognitive systems.

### Medication influence

**Fig. 3:**
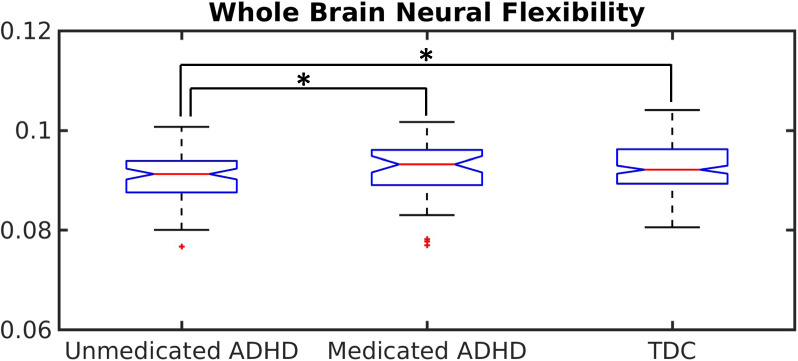
Influence of medication on neural flexibility in ADHD. A boxplot shows significantly increased whole brain neural flexibility in subjects with ADHD who are receiving treatment with medication as compared to unmedicated ADHD subjects (corrected *p* = 0.025), significantly decreased whole brain neural flexibility in unmedicated ADHD subjects as compared to the TDC group (corrected $p = 0.012$), no significant difference in whole brain neural flexibility between medicated ADHD subjects and subjects in the TDC group (corrected $p = 0.74$). Black asterisks indicate significant differences after FDR correction. Statistical significance levels: *p < 0.05.

A total of 46 subjects with ADHD received medication. Thus, we evaluated the influence of medication on neural flexibility in the ADHD group (Fig. 3). We found that whole brain neural flexibility was significantly higher in the medicated ADHD group than the unmedicated group (${{{{p}}}} = 0.025$, FDR corrected for three comparisons). Meanwhile, no statistical differences were observed between the medicated ADHD group and the TDC group (${{{{p}}}} = 0.74$, FDR corrected for three comparisons). Moreover, ADHD subjects in the unmedicated group exhibited significantly decreased neural flexibility when compared to TDC ($p = 0.012$, FDR corrected for three comparisons). Network-level and region-level analyses demonstrate these findings are largely consistent across brain networks and regions (Fig. S4, Table S6). Together, these results indicate treatment with medication has significant impact on the “recovery” of neural flexibility toward that observed in TDC subjects.

### Differentiating ADHD from TDC

We hypothesized that neural flexibility could serve as a biomarker to differentiate children with ADHD from TDC. Due to the smaller number of female subjects with ADHD, the observed sex differences in neural flexibility (Fig. S5), and the observed “recovery” of neural flexibility for subjects with ADHD on medication, we excluded both female and medicated ADHD subjects from training of the machine learning model. We performed ten-times tenfold CV, on the PKU dataset (TDC/ADHD: 65/51). The final model was then applied to the NYU dataset (TDC/ADHD: 31/16) as an independent test.

**Fig. 4:**
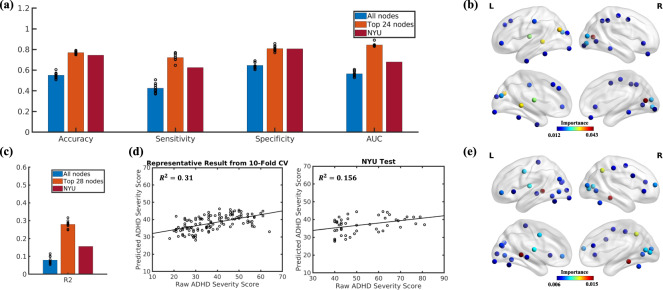
Successful prediction of ADHD status and severity using neural flexibility. **a** The accuracy, sensitivity, specificity, and AUC when using all 264 ROIs, top 24 ROIs, and independent testing using the NYU dataset with the top 24 ROIs, respectively for the ADHD classification model, and **b** the spatial distribution of the most predictive 24 regions using ranked importance scores. **c** The *R*^2^ scores when using all 264 ROIs, top 28 ROIs, and independent testing using the NYU dataset with the top 28 ROIs, respectively for the ADHD severity regression model, and **d** scatter plots comparing the representative neural flexibility-based severity score using tenfold cross validation and clinically obtained ADHD severity score using the top 28 regions for PKU dataset (left) and independent testing using the NYU dataset (right). **e** The spatial distribution of the most predictive 28 regions using ranked importance scores.

When including the neural flexibility of all 264 brain regions, an accuracy of 54.98% (sensitivity: 42.43%; specificity: 64.66%; and AUC: 56.46%) was achieved using the PKU dataset (Fig. 4a). Using the ranked importance scores, an optimal set of brain regions whose neural flexibility could better differentiate subjects with ADHD from TDC was identified (Fig. 4b and Fig. S6). When limiting the model to brain regions with the highest 24 importance scores, an accuracy of 77% (sensitivity: 72.13%; specificity: 80.78%; and AUC: 84.32%) was achieved (Fig. 4a, b). These 24 regions spanned 8 functional systems (VIS: 6 nodes; UC: 5 nodes; DMN and FPN: 4 nodes each; SAL and SH: 2 nodes each; SUB: 1 node) (Table S3). Lastly, we applied the PKU trained classification model with the 24 most predictive brain regions to the NYU dataset, and achieved an accuracy of 74.46% (sensitivity = 62.5%, specificity = 80.64% and AUC = 67.94%), demonstrating the robustness of the proposed approaches for differentiating ADHD from TDC.

### Neural flexibility-based ADHD score

We next evaluated the performance of the regression model by comparing the neural flexibility-based ADHD severity score and the clinically obtained ADHD severity score. Using neural flexibility of all 264 brain regions and the PKU dataset, the average *R*^2^ score from tenfold CV with ten repetitions was 0.079. In contrast, an average *R*^2^ of 0.2794 was achieved using the features selected from the brain regions with the highest 28 importance scores (Fig. 4c, d and Fig. S7). These 28 regions spanned 11 functional systems (VIS: 8 nodes; DMN: 4 nodes; FPN: 3 nodes; SH, SAL, SUB, DA and CB: 2 nodes each; VA, CO and UC: 1 node each). The spatial distribution of these regions is provided in Fig. 4e and details of these regions are summarized in Table S4. Applying this regression model and the selected 28 regions to the NYU dataset yielded an *R*^2^ of 0.156.

## Discussion

Extending research in ADHD indicating disruptions in dynamic FC [26–30], here we examined the alterations of neural flexibility in children with ADHD by employing a sliding window approach to estimate multilayer networks. Overall, we found that subjects with ADHD exhibited significantly decreased neural flexibility and altered dynamic modular structure (Fig. S1). These findings suggest that functional modules are less segregated in subjects with ADHD than in TDC, consistent with the previously reported impaired segregation of the default network and task-positive networks in ADHD [15]. Since neural flexibility has been associated with learning and executive functions [42, 44, 57], this decreased neural flexibility may underlie the compromised performance in the domain of executive function observed in ADHD.

### ADHD leads to a system-wise neural flexibility reconfiguration

Decreased neural flexibility was observed in both higher order networks and primary networks. Our results suggest a system-wide dynamic reconfiguration in ADHD rather than a disruption limited to specific sub-systems. Indeed, recent neurobiological models of ADHD have favored multi-network explanations, and the observed differences in functional organization as compared to TDC are widely distributed [13, 15, 58]. Furthermore, though dynamic network reconfiguration has been less investigated in ADHD, existing research is consistent with our observation of a system-wise reduction in neural flexibility. For instance, Rolls et al reported decreased temporal variability of the FC [30]. Another study reported that children with ADHD spent more time in a hyperconnected state as compared to TDC [26]. Duffy et al. further demonstrated that ADHD children with reduced temporal variability is related to higher commission errors using a go/no-go task, indicating the impaired executive functions of ADHD subjects [16]. Meanwhile, hyperconnectivity patterns were also widely reported in ADHD subjects [30, 58, 59], which would potentially “lock” regions together, constrain regional module transition frequency, and reduce neural flexibility.

Nevertheless, recent adult studies using the multilayer framework reported that adults with ADHD had higher flexibility and lower integration coefficient than of control subjects [60, 61], opposite to our findings. While several potential factors may explain the observed discrepancies, one of the most plausible reasons may be the difference in age across the studies (current cohort: mean age 11.65 years; adult cohorts: mean age 32 years). It has been widely documented that higher-order brain functions follow a protracted developmental timeline, well into adolescence or early adulthood [21, 62, 63]. Therefore, the observed discrepancies may reflect the complex developmental processes associated with ADHD. Future studies executing a direct comparison between different age groups and/or utilizing a longitudinal design are warranted.

### Medication effects

In this study, a total of 46 subjects were treated with stimulant medication. By comparing them to the unmedicated ADHD children, we found that medication use led to increased neural flexibility that was no longer significantly different from that observed in TDC. Since psychostimulant medications were withheld 24–48 h prior to scanning, the observed “recovery” of neural flexibility in the medication group may reflect the long-term benefit of stimulant medication to brain function. Consistent with our findings, previous structural and functional MR imaging studies also suggested that ADHD subjects who received stimulant treatment were more similar to TDC than unmedicated subjects with ADHD [46, 64]. These results suggest that neural flexibility is a sensitive metric revealing the alterations of intrinsic brain function in response to stimulant medication.

### Neural flexibility-based prediction

Machine learning methods have been increasingly employed to differentiate ADHD patients from TDC and predict clinical outcomes [8, 65]. Using the open access ‘ADHD-200’ dataset, a number of prediction models have been developed, with a range of accuracy from 55 to 90% [66–77]. Although, previous studies have reported promising prediction performance, concerns haven been raised about their methodological robustness [8]. Specifically, after reviewing 69 studies using neuroimaging features to predict ADHD diagnosis, Pulini et al. indicated that high classification accuracy appears to be inflated by circular analysis and small sample size and that many studies lack independent validation [78]. To mitigate these concerns, we combined tenfold CV and independent testing procedures in this study. First, ten repetitions of tenfold CV were applied. Since partitioning the dataset into tenfolds could yield random effects that may influence prediction performance, performing multiple repetitions should minimize this effect. Second, our models were validated using an independent dataset from NYU. Considering the experimental differences between these two datasets (imaging protocol, PKU: eye open/closed, NYU: eye closed, PKU: ADHD Rating Scale IV, and NYU: CPRS-LV), which could greatly influence the consistency of the data, our models still yield acceptable prediction performance.

Finally, aforementioned studies largely focused on accurate classification of ADHD. To date, only a few studies have been conducted to predict ADHD symptom severity [79]. In this study, a regression model was developed by identifying patterns of neural flexibility that are predictive of clinically obtained ADHD severity score ($R^2$: 0.2794 for tenfold CV, 0.156 for independent test). Currently, clinical ADHD diagnosis mainly relies on behavioral assessments after symptoms onset and have potential rater bias during implementation. Our findings could potentially inform efforts at earlier detection for vulnerable youth.

### Core regions of prediction

Using the ranked regional importance scores yielded by the XGBoost algorithm, we identified 24 brain regions that yielded the highest classification accuracy and 28 core regions that yielded the highest ${{{{{{{\mathrm{R}}}}}}}}^2$ for predicting ADHD severity. We refer to these brain regions as the core regions for classification (CR_c) and regression (CR_r) models. We found that models including only the core regions outperformed models including all regions. These findings indicate that using all brain regions likely include noise, inevitably leading to a negative impact on the performance of the models. As a result, the use of the importance scores yielded by the XGBoost offers a method to potentially remove noise while preserving the features that are important for model training.

Among the detected 24 CR_c and 28 CR_r, 11 regions (VIS: 4 nodes; DMN: 3 nodes; FPN: 2 nodes; SUB and UC: 1 node each) were consistently observed, suggesting that they may play key roles for delineating ADHD from TDC as well as for predicting ADHD severity. Indeed, using these 11 regions, comparable performance to the CR_c and CR_r models was achieved from ten times tenfold cross validation within the PKU dataset (${{{{{{{\mathrm{Accuracy}}}}}}}} = 71.43{{{{{{{\mathrm{\% }}}}}}}}$, ${{{{{{{\mathrm{R}}}}}}}}^2 = 0.23$), as well as from the independent testing set (${{{{{{{\mathrm{Accuracy}}}}}}}} = 70.21{{{{{{{\mathrm{\% }}}}}}}}$, ${{{{{{{\mathrm{R}}}}}}}}^2 = 0.11$). We also identified 13 regions specific to classification accuracy and 17 regions specific to regression accuracy. These regions were mainly located in DMN, FP, SAL, VIS, and attention networks. This indicates that while there is some overlap, there are also important differences between brain regions capable of distinguishing TDC and ADHD and predicting severity of ADHD symptoms.

### Limitations

There are several limitations of this study. First, as publicly available datasets, the imaging parameters and the rating systems of IQ and ADHD severity differ between PKU and NYU cohorts. Nevertheless, our prediction models were generalizable across the two cohorts. Second, we only considered males in the machine learning models due to the limited female subjects with ADHD. Third, we opted not to adjust IQ since the ADHD subjects tends to exhibit low IQ and controlling this variable can provide counterintuitive estimates of the effects of interest [11]. Finally, current study was performed with the limited sample size, the use of a cross-sectional design, and the use of data with short acquisition time. A longitudinal and prospective study with a larger sample size, longer scanning time and detailed medication information will be needed to further confirm our findings.

## Conclusion

In conclusion, we investigated the dynamic functional network reconfiguration in ADHD. Significantly decreased neural flexibility was observed in children with ADHD spanning multiple brain functional networks, supporting the multi-network explanations of ADHD. Using the XGBoost approach, core regions critically important for differentiating ADHD from TDC, as well as predicting ADHD severity were reported. Finally, we were able to successfully classify group membership and predict ADHD severity using an independent testing dataset, demonstrating the robustness of these approaches. Our study demonstrated the potential clinical utility of neural flexibility to diagnose children with ADHD and monitor disease severity.

## Supplementary information

SUPPLEMENTAL MATERIAL
