# Supplementary material for: Altered neural flexibility in children with attention-deficit/hyperactivity disorder
Source: Mol Psychiatry. 2022 Jul 22;27(11):4673–9. doi: 10.1038/s41380-022-01706-4 (PMC9734048; doi:10.1038/s41380-022-01706-4)
Supplement: Supplementary file 1 — SUPPLEMENTAL MATERIAL [file 41380_2022_1706_MOESM1_ESM.docx]

**Supplementary Information**

**Supplementary Methods**

***Data information***

We used a publicly available ADHD dataset, ADHD-200 study, from Peking University (PKU) and New York University (NYU), with 236 and 192 subjects, respectively. For the PKU dataset, ADHD was initially assessed using the Computerized Diagnostic Interview Schedule IV (C-DIS-IV) ^1^ and further verified with the Schedule of Affective Disorders and Schizophrenia for Children—Present and Lifetime Version (KSADS-PL) ^2^. For the NYU dataset, ADHD was evaluated with KSADS-PL and Conner’s Parent Rating Scale-Revised, Long version (CPRS-LV) ^3^. ADHD indices from ADHD Rating Scale IV^4^ (PKU) and CPRS-LV (NYU) were obtained, reflecting clinically assessed ADHD severity of individuals. Intelligence (IQ) was assessed with Wechsler Intelligence Scale for Chinese Children-Revised^5^ (PKU) and Wechsler Abbreviated Scale of Intelligence^6^ (NYU), respectively. Exclusion criteria included left-handedness, IQ below 80, no ADHD index, loss of consciousness due to head trauma, neurological illness, schizophrenia, affective disorder, pervasive development disorder, substance abuse. Additionally, subjects failed to pass quality control of image preprocessing were also excluded from the analysis, including no full brain coverage, failed tissue segmentation, failed image registration, and excess motion (mean FD > 0.3 mm, maximal head motion of more than 5mm or 5 degrees). Detailed imaging parameters can be found from ADHD-200 website ( <http://fcon_1000.projects.nitrc.org/indi/adhd200/> ) and summarized in Table S1.

***Preprocessing***

RsfMRI were preprocessed using FSL, which included discarding the first 10 volumes, slice-timing correction, motion correction, spatial smoothing (6mm full-width at half-maximum), bandpass filtering (0.01 Hz ~ 0.08 Hz), global mean/white matter/cerebrospinal fluid (CSF) signal regression, 24 head motion parameters regression and wavelet denoising ^7, 8^. The time series lengths varied among subjects and imaging sites. To minimize biases contributed by the varying lenghts of time series data, the total time series length was kept at 225 for PKU subjects and 165 for NYU. For each subject, T1-weighted images were first segmented into three tissue types, including gray and white matter and CSF. The tissue segmentation images were then normalized to a standard template using the advanced normalization tools (ANTs) ^9^. Using the deformation field, the Power264 atlas ^10^ was deformed back to rsfMRI space to extract the mean time series of each ROI. Specifically, Power264 atlas parcellated the brain into 264 regions and 13 functional systems, including sensorimotor hand (SH), sensorimotor mouth (SM), auditory (AUD), visual (VIS), cingulo-opercular (CO), frontoparietal (FP), default model (DMN), memory retrieval (MEM), salience (SAL), subcortical (SUB), ventral attention (VA), dorsal attention (DA), cerebellar (CB), as well as an uncertain system (UC).

***Multilayer community detection***

The multilayer networks constructed according to the main text include weighted edges representing statistically significant correlations within each temporal layer along with interlayer coupling connections connecting each node (ROI) to itself in the adjacent time layers. Dynamic community detection was performed on each multilayer network using the Generalized Louvain method ^11, 12^, which has been employed to study the dynamic community structure in time-dependent, multilayer, and multiplex networks. The quality function (Q) is defined as:

$$Q=\frac{1}{2\mu}\sum_{ijsr} \left[ \left( A_{ijs}-\gamma_{s}\frac{\kappa_{is}\kappa_{js}}{2m_{s}} \right)\delta_{sr}+\delta_{ij}\omega_{jsr} \right]\delta\left( g_{is},g_{jr} \right)$$

where the (weighted) adjacency matrix of layer $s$ has components $A_{ijs}$; $\gamma_{s}$ is the resolution parameter of layer $s$; $g_{is}$ and d $g_{jr}$ are the community assignment of node $i$ in layer $s$ and node $j$ in layer $r$, respectively; $\omega_{jsr}$ is the interlayer coupling strength parameter between node $j$ in layer $r$ and layer $s$; $\mu$ is the total edge weight in the network and calculated as $\mu=\frac{1}{2}\sum_{jr} \kappa_{jr}$; $k_{js}=\sum_{i} A_{ijs}$is the intra-layer strength of node $j$ in layer $s$ and; the inter-layer strength of node $j$ in layer $s$ is $c_{js}=\sum_{r} \omega_{jsr}$; the strength of node $j$ in layer $s$ is $\kappa_{js}=k_{js}+c_{js}$ ; $m_{s}$ is the total edge weight in layer $s$ and defined as $m_{s}=\frac{1}{2}\sum_{ij} A_{ijs}$. In our analysis here, the layer here refers to the (statistically significant elements of the) correlation coefficient matrix of a given sliding window in time.

In this study, the primary parameters for the Generalized Louvain algorithm were chosen identical to the reference ^13, 14^ (i.e. $\gamma=1, \omega=1$). However, the choice of $\gamma$ and $\omega$ could influence community detection results, leading us to consider alternative values for comparison. While smaller (larger) $\gamma$ typically result in larger (smaller) modules, different inter-layer coupling strengths (i.e. $\omega$) affect the detected dynamic structures by making it easier (lower $\omega$) or harder (higher $\omega$) for nodes to switch community labels through time. Therefore, it is important to test the stability of neural flexibility patterns across different choices of γ and ω. We thus conducted additional analysis (see below in “*Effects of the resolution and coupling parameters on neural flexibility*”) considering values of γ and ω in the range from 0.5 to 2.

Dynamic community structure in the brain can also be usefully characterized using module number, module size and module stationarity. Specifically, module number was defined as the total number of modules across all time windows. The mean module size was defined as the average number of ROIs per module in a time window, averaged across all of the time windows that a given module exists. The stationarity of module ^15^ was defined as:

$$\zeta=\frac{\sum_{t=t_{0}}^{t^{'}-1} U\left( t,t+1 \right)}{t^{'}-t_{0}-1}$$

where $t_{0}$ is the time when the module appears and $t^{'}$ the time before the module disappears, and $U$ is the autocorrelation function of the module $G\left( t \right)$ between consecutive time points:

$$U\left( t,t+1 \right)=\frac{\left| G\left( t \right)\cap G\left( t+1 \right) \right|}{\left| G\left( t \right)\cup G\left( t+1 \right) \right|}$$

where $\left| G\left( t \right)\cap G\left( t+1 \right) \right|$ is the number of nodes that are members of both $G\left( t \right)$ and $G\left( t+1 \right)$, while $\left| G\left( t \right)\cup G\left( t+1 \right) \right|$ is the total number of nodes in $G\left( t \right)$ and $G\left( t+1 \right)$.

***Regression analysis***

In this study, linear regression was applied to evaluate statistical differences between groups (e.g. ADHD vs TDC, unmedicated ADHD vs medicated ADHD, unmedicated ADHD vs TDC, medicated ADHD vs TDC), including age, sex, imaging site, and mean FD as covariates. The regression model is:

$F=B_{0}+B_{1}\left( Age \right)+B_{2}\left( Sex \right)+B_{3}\left( Site \right)+B_{4}(FD)+B_{5}(Group Label)$ .

Here, $F$ is the response variable, the neural flexibility. The group label can be generated based on ADHD vs TDC, unmedicated ADHD vs medicated ADHD, unmedicated ADHD vs TDC, and medicated ADHD vs TDC.

***XGBoost***

The extreme gradient boosting (XGBoost) is an ensemble learning algorithm where multiple decision trees were constructed to solve both classification and regression problems ^16^. It has been widely used to provide accurate performance across numerous applications ^17-21^. The ensemble learning algorithm combines multiple learners together to provide better predictive performance than that obtained using a single learner alone ^22^. Furthermore, boosting theory constructs the final model in an adaptive way via fitting multiple learners sequentially; each learner in the sequence is fitted by giving more importance to the observations in the dataset that were badly handled by the previous learners in the sequence. In other words, each new learner focuses its efforts on the most difficult observations to reduce the bias of the model. Decision trees have been widely used as a basic learner in different ensemble learning algorithms, such as XGBoost and random forest. It uses a tree-like model to go from features about an item (represented in the branches) to conclusions about the item’s target value (represented in the leaves). Leaves can represent class labels or continuous values for classification/regression type of problems, respectively.

In this study, we first trained and validated the performance of models on the PKU dataset, and then applied the models to the NYU dataset to independently test the performance of the trained models in an out-of-sample, cross-dataset setting. Our classification and regression models each followed four common steps: (I) model training, (II) feature ranking and selection, (III) performance evaluation on validation dataset, and (IV) performance evaluation using the independent NYU dataset.

For the training and validation processes, we used 10-fold cross validation randomly repeated 10 times. Each time, nodal neural flexibility measures from 264 regions and the corresponding ADHD labels/indices from nine-folds training subjects were used to train the models, and the remaining one-fold data were used to validate the performance. These procedures were repeated 10 times with different splits of the data to avoid the influence of random division.

The XGBoost algorithm ranks the importance of each feature and provides an “importance score.” Specifically, the more frequent a feature is used to make key decisions in the model, the higher its importance score becomes. Thus, brain regions playing important roles for classification/regression models can be revealed by ranking their importance scores among all brain regions. We first considered all 264 brain regions for both the classification and regression models. Subsequently, we attempted to determine if an optimal combination of brain regions can be obtained to improve performance of the models. Specifically, we evaluated the performance of the top N ($N\in[1,264]$) regions based on their averaged importance scores from our 10 times 10-fold cross validations and determined a set of brain regions that yielded the optimal performance for classification and regression models, separately.

The number of estimators, subsample ratio of training instances, subsample ratio of columns when constructing each tree, max depth of a tree, and learning rate were tuned to find the optimal values, since these parameters have major impact to the model performance. We picked the hyperparameters based on the model performance from optimal ROIs that were detected as the subset of regions reaching ideal performance. Other parameters were chosen as default values from Python XGBoost package (version 1.2.0). The hyperparameters used in this study were summarized in Table S2.

Finally, again, ADHD and TDC subjects from the NYU dataset were used for independent testing. Specifically, the classification and regression models trained on the full PKU data were applied to the NYU data to assess the out-of-sample robustness of models.

**Supplementary Results**

***Brain dynamic modular structure***

We observed not-statistically-significant differences in multilayer modularity between ADHD and TDC subjects (raw $p=0.61$) (Figure S1a). However, the number of modules of ADHD subjects was significantly lower than that of the TDC group (raw$p=8.6\times{10}^{-4}$) (Figure S1b). A significantly increased modular size was also observed in ADHD group (raw $p=0.018$) (Figure S1c). We note in particular that, because of the nodes-by-time nature of the community labels, a lower number of modules in total across all time layers does not by itself necessitate larger average module size per layer. Finally, a higher module stationarity was found in ADHD subjects (raw $p=0.0085$) (Figure S1d), suggesting that the functional modules of ADHD subjects were more temporally stable than that of the TDC group.

These findings suggest that the brain functional modules are less segregated in ADHD subjects than those in the TDC group, consistent with the previously reported impaired segregation of the default network and task-positive networks in ADHD ^23^. A significantly increased module stationarity was observed, indicating a higher module stability across time. This finding is consistent with the reported decreased variability in ADHD ^24^.

***Regional-level analysis of neural flexibility***

Of all the 264 regions, 55 regions spanned over 13 networks showed a decreased neural flexibility in ADHD subjects when compared to that of TDC (raw $p<0.05$) (Figure S2a, Table S5). Of the 55 regions, the sensory hand network exhibits the largest number of regions with altered neural flexibility (12 regions), followed by the visual network (7 regions), salience network (7 regions), subcortical network (6 regions), and default network (6 regions) (Figure S2b). These findings are consistent with the system-level analysis, suggesting that ADHD subjects exhibited reduced neural flexibility spanning across multiple functional networks encompassing both basic and higher order cognitive systems. However, due to the limited sample size, only four regions (ROI 15: middle cingulate gyri, ROI 20: supramarginal gyrus, ROI 23: postcentral gyrus, ROI 120: middle temporal gyrus) could pass FDR correction.

Among these 55 regions, 9 regions were overlapped with the detected core regions of classification model (24 regions) and regression model (28 regions), respectively. Clearly, these findings suggest the inconsistency between statistically important regions and data-driven selected core regions. Of note, in this study, the statistically determined important regions were obtained from both cohorts, including both medicated and unmedicated ADHD subjects, while data-driven selected core regions were picked from the PKU site data by excluding medicated subjects. Site and medication effect would influence the statistical results. Meanwhile, the data-driven approach tends to select uncorrelated features to reduce feature redundancy and improve prediction performance. Therefore, some regions, though not associated with significantly difference between groups, were selected.

Among the detected core regions, 11 regions (VIS: 4 nodes; DMN: 3 nodes; FPN: 2 nodes; SUB and UC: 1 node each) were consistently observed. We further evaluated the inter-regional correlation of these 11 core regions (Figure S3a), as well as the probability of these core regions being assigned to the same module across time and subjects (Figure S3b). Three regions were observed to have strong correlations with each other (ROI 145 [Calcarine], ROI 148 [Lingual], and ROI 167 [Cuneus]). Furthermore, there was approximately 50% probability that these regions were assigned to the same modules. The remaining 8 regions were less correlated with each other or not likely to be assigned to the same modules. Together, these results suggested that these three regions are highly inter-correlated, while the eight remaining regions are potentially independent of each other.

***Medication influence on neural flexibility at system and regional levels***

Additional analysis was performed to evaluate the medication influence at network and regional levels. As shown in Figure S4a, a general pattern showing an increased neural flexibility in the medication treated group when compared to the medication naïve group was observed. Specifically, the neural flexibility of FPN and DA networks were significantly increased in the medication treated group (raw $p<0.05$). However, due to the limited sample size, none of them could pass FDR correction. Of all the 264 regions, 14 regions spanned over 7 networks showed an increased neural flexibility in the medication treated group when compared to the medication naïve group (raw $p<0.05$) (Table S6). None of them could pass FDR correction. Together, these results indicate the general recovery pattern of neural flexibility with medication towards that observed in TDC.

Figure S4b further shows the results comparing neural flexibility of medicated ADHD subjects with the TDC group. No statistical differences were observed in all the functional systems (raw $p>0.05$). Of the 264 regions, only 7 regions showed neural flexibility difference between groups (raw $p<0.05$) (Table S6). After FDR correction, none of them exhibited significant difference between medicated ADHD subjects and TDC.

Nevertheless, current analysis is limited by the cross-sectional study design and sample size. Further studies with larger sample size and longitudinal study design will be required to confirm our findings.

***Sex influence***

A total of 84 female TDC samples (PKU/NYU: 46/38) and 35 female ADHD samples (PKU/NYU: 9/26) were included in the statistical analysis. We found that whole brain neural flexibility was significantly decreased (raw $p=0.0008$) in the female subjects when compared to that in the male subjects (Figure S5). Besides, only 16 female ADHD samples were under confirmed medication naïve status. In other words, including female samples in the machine learning models would introduce unbalanced sex distribution in TDC (M/F: 96/84) and ADHD groups (M/F: 67/16), which could influence the model performance. In addition, according to the Centers for Disease Control and Prevention ( <https://www.cdc.gov/ncbddd/adhd/data.html> ), males are two times more likely to be diagnosed with ADHD than females (12.9% vs 5.6%). Therefore, considering the limited female samples, the observed neural flexibility difference, as well as the higher diagnosis rate in males, our machine learning models focused on male subjects. Nevertheless, future work with sufficient female samples should help to optimize the models.

***Effects of templates***

Recent study has indicated the anatomical difference between Caucasian populations and Chinese populations, especially in the lateral frontal and parietal regions ^25^. Therefore, assessing the potential influence of using different brain templates may be necessary. Here, we tested the robustness of our findings by changing the template to the Chinese pediatric template (CHN-PD) and Caucasian pediatric template (NIH-PD) ^25, 26^. Specifically, the PKU subjects were registered to CHN-PD, and the NYU subjects were registered to NIH-PD, respectively. Since the Power264 atlas was defined in the adult MNI space, additional ANTs registrations between the pediatric templates and the adult MNI template were performed to warp the Power264 atlas to each pediatric template space, respectively.

We found that the use of pediatric templates yielded results consistent with those reported in the main text. A significantly decreased whole brain neural flexibility was observed in ADHD compared to TDC (raw $p=0.0008$) when using pediatric template (Figure S8a). Moreover, no statistical difference of whole brain neural flexibility (Figure S8b) nor in the ROI-level analysis (raw $p>0.05$) were observed between using adult MNI and pediatric specific templates. Additionally, we compared the spatial similarity of the distribution of neural flexibility by calculating the Pearson’s correlation of neural flexibility across ROIs as calculated with using pediatric templates and adult MNI templates. A high spatial similarity ($R>0.98$) of the distribution of neural flexibility was observed, indicating that the spatial patterns of neural flexibility are highly consistent across the use of different templates (Figure S8c).

***Effects of network thresholding strategies***

In the main text, a statistical significance thresholding strategy was performed to remove weak and spurious connections, resulting in differences in network density across sliding windows and across subjects. Though this approach was widely applied in previous multilayer network studies to construct functional connectivity matrices ^13, 14, 27, 28^, it is of importance to evaluate the robustness of our findings with a different network thresholding strategy. To this end, we evaluated our results using a fixed network layer edge density of 10%, which is a common threshold used in previous studies ^29-31^. Consistent with the results reported in the main text, the while brain neural flexibility in ADHD was significantly reduced when compared to the TDC group (raw $p=0.04$) (Figure S9a). Furthermore, we compared the spatial similarity of the obtained neural flexibility between a fixed network density strategy and a statistical significance thresholding strategy among sites and groups. A spatial similarity over 0.72 was obtained (Figure S9b), indicating that the spatial patterns of neural flexibility are highly similar between the two thresholding strategies.

***Effects of the resolution and coupling parameters on neural flexibility***

When assessing the community detection results, one always encounters the questions about the proper setting of the method parameters. In the multilayer modularity community detection method, the resolution parameter γ and coupling parameter ω would influence the community detection results. Specifically, by considering multiple values of γ, we may reveal different module sizes ^32, 33^, i.e. smaller γ typically result in larger modules whereas larger γ yields smaller modules. Meanwhile, different choices of the coupling parameter ω also influence the detected dynamic structures ^11^. In this study, we chose $\gamma=1, \omega=1$ as our primary parameters since these are typically taken as defaults and have been widely used in previous studies to provide meaningful results ^14, 34-37^. However, it is important to test the stability of our results based on neural flexibility patterns across different choices of γ and ω. Here, we conducted additional analysis by considering γ and ω from 0.5 to 2.

As expected, the absolute values of neural flexibility vary with the choices of γ and ω. However, the whole brain neural flexibility in ADHD continued to be significantly reduced when compared to the TDC group across a wide range of γ and ω (γ from 0.75 to 2, $\omega$ from 0.5 to 2) (Figure S10). In addition, a high spatial similarity of the distribution of neural flexibility was also revealed within this γ and ω range (Figure S11), indicating a highly stable spatial distribution of neural flexibility across different community detection parameters.

***Effects of spatial smoothing on neural flexibility***

Spatial smoothing was commonly applied to increase signal-to-noise ratio, which has been widely used in the pediatric and adult fMRI preprocessing pipelines ^7, 29, 38^. However, the necessity of spatial smoothing in ROI-based analysis is still under debate. To evaluate the robustness of our findings, we conducted additional analysis without spatial smoothing step in preprocessing.

Even though the absolute values of neural flexibility were changed, consistent with the findings reported in the manuscript, a significantly decreased pattern of whole brain neural flexibility in ADHD subjects was observed when compared to that of the TDC group (Figure S12a). Furthermore, we compared the spatial similarity of the distribution of brain neural flexibility between using 6mm spatial smoothing and without smoothing. Spatial similarity over 0.79 was obtained (Figure S12b), indicating that the spatial distribution patterns of neural flexibility are highly similar with and without smoothing.

***Effects of the window length for the measure of neural flexibility***

It is necessary to choose a window length when performing the sliding window approach to define the multilayer network representation of the temporal data. If the window length is too long, the ability to estimate dynamic features will be compromised. Conversely, if the window length is too short, the statistical power for estimating functional connectivity will be reduced. Recent simulation and human studies have reported that a window length of 40s-100s could minimize spurious dynamics ^39-44^. In the main text, we reported the results using a window length of 60 seconds, which is within the suggested range. Nevertheless, to further evaluate whether the choice of window length may affect the conclusions of our study, we conducted additional analyses using two additional window lengths, 40 and 80 sec, respectively.

Even though the absolute values of neural flexibility varied with different window lengths, a significantly decreased pattern of whole brain neural flexibility was consistently observed in the ADHD group when compared to the TDC group (Figure S13a-c). Furthermore, we further compared the spatial distribution of neural flexibility using a window length of 40s, 60s, and 80s, respectively. A spatial similarity over 0.9 was obtained using different window lengths (Figure S13d-e), indicating that the spatial patterns of neural flexibility are highly similar over different window lengths.

**Supplementary Tables**

**Table S1. Imaging parameters of the ADHD dataset**

|  |  |  | ***PK_1*** | ***PK_2*** | ***PK_3*** | ***PK_1_test*** |
| --- | --- | --- | --- | --- | --- | --- |
| ***PKU*** | ***Anatomical*** | TR/TE | 2530/3.39 | 2530/3.45 | 5 protocols* | 2530/3.39 |
|  |  | Slices | 128 | 176 | 5 protocols* | 128 |
|  |  | Thickness(mm) | 1.33 | 1 | 5 protocols* | 1.33 |
|  |  | Flip angel(degree) | 7 | 7 | 5 protocols* | 7 |
|  |  |  |  |  |  |  |
|  | ***fMRI*** | TR/TE | 2000/30 | 2000/30 | 2000/30 | 2000/30 |
|  |  | Slices | 33 | 33 | 30 | 33 |
|  |  | Thickness(mm) | 3.5 | 3 | 4.5 | 3.5 |
|  |  | Flip angel(degree) | 90 | 90 | 90 | 90 |
|  |  | Volumes | 240 | 240 | 240 | 239 |
|  | | | | | | |
|  |  |  |  | | | |
| ***NYU*** | ***Anatomical*** | TR/TE | 2530/3.25 | | | |
|  |  | Slices | 128 | | | |
|  |  | Thickness(mm) | 1.33 | | | |
|  |  | Flip angel(degree) | 7 | | | |
|  |  |  |  | | | |
|  | ***fMRI*** | TR/TE | 2000/15 | | | |
|  |  | Slices | 33 | | | |
|  |  | Thickness(mm) | 4 | | | |
|  |  | Flip angel(degree) | 90 | | | |
|  |  | Volumes | 176 | | | |

*PK_1, PK_2, PK_3 and PK_1_test are four subgroups of PKU cohort. Additional details can be found in <http://fcon_1000.projects.nitrc.org/indi/adhd200/>

**Table S2. XGBoost parameters***

|  | **n_estimators** | **subsample** | **colsample_bytree** | **max_depth** | **learning_rate** |
| --- | --- | --- | --- | --- | --- |
| ***Classification model*** | 210 | 0.9265 | 0.3387 | 1 | 0.3 |
| ***Regression model*** | 50 | 1 | 0.3387 | 7 | 1 |

*****Other parameters were chosen as default values from the python XGBoost package (version 1.2.0).

**Table S3. Top 24 regions for ADHD classification**

| **ROI** | **MNI Space** | | | **Name** | **System** |
| --- | --- | --- | --- | --- | --- |
|  | **X** | **Y** | **Z** |  |  |
| 4 | -56 | -45 | -24 | Inferior temporal gyrus | Uncertain |
| 5 | 8 | 41 | -24 | Gyrus rectus | Uncertain |
| 20 | -54 | -23 | 43 | Supramarginal gyrus | Sensorimotor hand |
| 36 | 42 | -20 | 55 | Precentral gyrus | Sensorimotor hand |
| 74 | -41 | -75 | 26 | Middle occipital gyrus | Default mode |
| 77 | -13 | -40 | 1 | Lingual gyrus | Default mode |
| 112 | -2 | 38 | 36 | Superior frontal gyrus, medial | Default mode |
| 125 | 27 | -37 | -13 | Fusiform gyrus | Default mode |
| 140 | 8 | -91 | -7 | Lingual gyrus | Uncertain |
| 142 | -12 | -95 | -13 | Lingual gyrus | Uncertain |
| 145 | 8 | -72 | 11 | Calcarine fissure | Visual |
| 148 | 20 | -66 | 2 | Lingual gyrus | Visual |
| 149 | -24 | -91 | 19 | Middle occipital gyrus | Visual |
| 158 | 20 | -86 | -2 | Lingual gyrus | Visual |
| 167 | -3 | -81 | 21 | Cuneus | Visual |
| 169 | 37 | -84 | 13 | Middle occipital gyrus | Visual |
| 190 | 49 | -42 | 45 | Supramarginal gyrus | Frontoparietal |
| 198 | -42 | 45 | -2 | Middle frontal gyrus, orbital part | Frontoparietal |
| 199 | 33 | -53 | 44 | Angular gyrus | Frontoparietal |
| 202 | -3 | 26 | 44 | Superior frontal gyrus, medial | Frontoparietal |
| 205 | 42 | 0 | 47 | Precentral gyrus | Salience |
| 210 | 37 | 32 | -2 | Inferior frontal gyrus, orbital part | Salience |
| 223 | -2 | -13 | 12 | Thalamus | Subcortical |
| 250 | -50 | -7 | -39 | Inferior temporal gyrus | Uncertain |

**Table S4. Top 28 Regions for generating neural flexibility-based ADHD severity score**

| **ROI** | **MNI Space** | | | **Name** | **System** |  |
| --- | --- | --- | --- | --- | --- | --- |
|  | **X** | **Y** | **Z** |  |  |  |
| 34 | -21 | -31 | 61 | Postcentral gyrus | Sensorimotor hand | |
| 51 | -10 | -2 | 42 | Median cingulate | Cingulo-opercular |  |
| 74 | -41 | -75 | 26 | Middle occipital gyrus | Default mode |  |
| 77 | -13 | -40 | 1 | Lingual gyrus | Default mode |  |
| 122 | 12 | 36 | 20 | Anterior cingulate | Default mode |  |
| 125 | 27 | -37 | -13 | Fusiform gyrus | Default mode |  |
| 142 | -12 | -95 | -13 | Lingual gyrus | Uncertain |  |
| 145 | 8 | -72 | 11 | Calcarine fissure | Visual |  |
| 147 | -28 | -79 | 19 | Middle occipital gyrus | Visual |  |
| 148 | 20 | -66 | 2 | Lingual gyrus | Visual |  |
| 164 | -42 | -74 | 0 | Middle occipital gyrus | Visual |  |
| 167 | -3 | -81 | 21 | Cuneus | Visual |  |
| 169 | 37 | -84 | 13 | Middle occipital gyrus | Visual |  |
| 170 | 6 | -81 | 6 | Calcarine fissure | Visual |  |
| 171 | -26 | -90 | 3 | Middle occipital gyrus | Visual |  |
| 186 | 47 | 10 | 33 | Precentral gyrus | Frontoparietal |  |
| 198 | -42 | 45 | -2 | Middle frontal gyrus, orbital part | Frontoparietal |  |
| 199 | 33 | -53 | 44 | Angular gyrus | Frontoparietal |  |
| 208 | -35 | 20 | 0 | Insula | Salience |  |
| 218 | 31 | 56 | 14 | Middle frontal gyrus | Salience |  |
| 223 | -2 | -13 | 12 | Thalamus | Subcortical |  |
| 226 | -5 | -28 | -4 | Thalamus | Subcortical |  |
| 241 | 53 | 33 | 1 | Inferior frontal gyrus, triangular part | Ventral attention |  |
| 243 | -16 | -65 | -20 | Cerebellum 6 | Cerebellar |  |
| 245 | 22 | -58 | -23 | Cerebellum 6 | Cerebellar |  |
| 255 | 47 | -30 | 49 | Postcentral gyrus | Sensorimotor hand |  |
| 262 | -42 | -60 | -9 | Inferior temporal gyrus | Dorsal attention |  |
| 264 | 29 | -5 | 54 | Precentral gyrus | Dorsal attention |  |

**Table S5. Regions with altered neural flexibility between ADHD and TDC (raw** $\boldsymbol{p<0.05}$**)**

| **ROI** | **MNI Space** | | | **P value (raw)** | **Name** | **System** |
| --- | --- | --- | --- | --- | --- | --- |
|  | **X** | **Y** | **Z** |  |  |  |
| 12 | 34 | 38 | -12 | 0.005071 | Inferior frontal gyrus, orbital part | Uncertain |
| 14 | -14 | -18 | 40 | 0.00482 | Middle cingulate | Sensorimotor hand |
| **15** | **0** | **-15** | **47** | **0.000669** | **Middle cingulate** | **Sensorimotor hand** |
| 17 | -17 | -21 | 65 | 0.034418 | Paracentral lobule | Sensorimotor hand |
| 18 | -7 | -33 | 72 | 0.013476 | Paracentral lobule | Sensorimotor hand |
| 19 | 13 | -33 | 75 | 0.030991 | Postcentral gyrus | Sensorimotor hand |
| **20** | **-54** | **-23** | **43** | **0.000539** | **Supramarginal gyrus** | **Sensorimotor hand** |
| **23** | **-23** | **-30** | **72** | **5.26E-05** | **Postcentral gyrus** | **Sensorimotor hand** |
| 24 | -40 | -19 | 54 | 0.013141 | Precentral gyrus | Sensorimotor hand |
| 26 | 50 | -20 | 42 | 0.048947 | Postcentral gyrus | Sensorimotor hand |
| 28 | 20 | -29 | 60 | 0.040911 | Precentral gyrus | Sensorimotor hand |
| 33 | -45 | -32 | 47 | 0.043183 | Postcentral gyrus | Sensorimotor hand |
| 34 | -23 | -31 | 61 | 0.032674 | Postcentral gyrus | Sensorimotor hand |
| 42 | -49 | -11 | 35 | 0.036667 | Postcentral gyrus | Sensorimotor mouth |
| 44 | 51 | -6 | 32 | 0.014419 | Postcentral gyrus | Sensorimotor mouth |
| 46 | 66 | -8 | 25 | 0.001373 | Postcentral gyrus | Sensorimotor mouth |
| 51 | -10 | -2 | 42 | 0.019819 | Middle cingulate | Cingulo-opercular |
| 61 | 32 | -26 | 13 | 0.007456 | Heschl gyrus | Auditory |
| 66 | -49 | -26 | 5 | 0.031532 | Superior temporal gyrus | Auditory |
| 73 | -30 | -27 | 12 | 0.021241 | Heschl gyrus | Auditory |
| 77 | -13 | -40 | 1 | 0.004594 | Lingual gyrus | Default mode |
| 112 | -2 | 38 | 36 | 0.003797 | Superior frontal gyrus, medial | Default mode |
| 113 | -3 | 42 | 16 | 0.021999 | Anterior cingulate | Default mode |
| 119 | 65 | -31 | -9 | 0.011042 | Middle temporal gyrus | Default mode |
| **120** | **-68** | **-41** | **-5** | **0.000596** | **Middle temporal gyrus** | **Default mode** |
| 139 | 49 | 35 | -12 | 0.036672 | Inferior frontal gyrus, orbital part | Default mode |
| 142 | -12 | -95 | -13 | 0.009727 | Lingual gyrus | Uncertain |
| 145 | 8 | -72 | 11 | 0.014398 | Calcarine fissure | Visual |
| 146 | -8 | -81 | 7 | 0.032841 | Calcarine fissure | Visual |
| 151 | -15 | -72 | -8 | 0.021733 | Lingual gyrus | Visual |
| 160 | -16 | -52 | -1 | 0.029505 | Lingual gyrus | Visual |
| 167 | -3 | -81 | 21 | 0.021165 | Cuneus | Visual |
| 170 | 6 | -81 | 6 | 0.004485 | Calcarine fissure | Visual |
| 173 | 37 | -81 | 1 | 0.023662 | Middle occipital gyrus | Visual |
| 174 | -44 | 2 | 46 | 0.016169 | Precentral gyrus | Frontoparietal |
| 181 | 34 | 54 | -13 | 0.043516 | Middle frontal gyrus, orbital part | Frontoparietal |
| 202 | -3 | 26 | 44 | 0.005968 | Superior frontal gyrus, medial | Frontoparietal |
| 208 | -35 | 20 | 0 | 0.002495 | Insula | Salience |
| 210 | 37 | 32 | -2 | 0.041088 | Inferior frontal gyrus, orbital part | Salience |
| 212 | -11 | 26 | 25 | 0.004701 | Anterior cingulate | Salience |
| 213 | -1 | 15 | 44 | 0.049721 | Supplementary motor area | Salience |
| 215 | 0 | 30 | 27 | 0.014455 | Anterior cingulate | Salience |
| 216 | 5 | 23 | 37 | 0.013834 | Middle cingulate | Salience |
| 217 | 10 | 22 | 27 | 0.0254 | Anterior cingulate | Salience |
| 221 | 2 | -24 | 30 | 0.035777 | Middle cingulate | Memory retrieval |
| 222 | 6 | -24 | 0 | 0.028788 | Thalamus | Subcortical |
| 223 | -2 | -13 | 12 | 0.00269 | Thalamus | Subcortical |
| 224 | -10 | -18 | 7 | 0.003517 | Thalamus | Subcortical |
| 225 | 12 | -17 | 8 | 0.003075 | Thalamus | Subcortical |
| 228 | -15 | 4 | 8 | 0.019197 | Caudate nucleus | Subcortical |
| 234 | 9 | -4 | 6 | 0.006215 | Thalamus | Subcortical |
| 235 | 54 | -43 | 22 | 0.020644 | Superior temporal gyrus | Ventral attention |
| 237 | -55 | -40 | 14 | 0.024978 | Superior temporal gyrus | Ventral attention |
| 252 | -52 | -63 | 5 | 0.001201 | Middle temporal gyrus | Dorsal attention |
| 257 | 46 | -59 | 4 | 0.020491 | Middle temporal gyrus | Dorsal attention |

**Table S6. Medication influence at regional level (raw** $\boldsymbol{p<0.05}$**).**

| **Medicated – Medicated Naïve** | | | | | | | |
| --- | --- | --- | --- | --- | --- | --- | --- |
| **ROI** | **MNI Space** | | | **P value (raw)** | **Difference** | **Name** | **Network** |
|  | **X** | **Y** | **Z** |  |  |  |  |
| 5 | 8 | 41 | -24 | 0.014 | 0.006 | Gyrus rectus | Uncertain |
| 36 | 42 | -20 | 55 | 0.012 | 0.008 | Precentral gyrus | Sensorimotor hand |
| 53 | 13 | -1 | 70 | 0.023 | 0.005 | Supplementary motor area | Cingulo-opercular |
| 112 | -2 | 38 | 36 | 0.01 | 0.006 | Superior frontal gyrus, medial | Default mode |
| 125 | 27 | -37 | -13 | 0.003 | 0.008 | Fusiform gyrus | Default mode |
| 143 | 18 | -47 | -10 | 0.048 | 0.007 | Lingual gyrus | Visual |
| 148 | 20 | -66 | 2 | 0.008 | 0.01 | Lingual gyrus | Visual |
| 152 | -18 | -68 | 5 | 0.034 | 0.008 | Calcarine fissure | Visual |
| 160 | -16 | -52 | -1 | 0.015 | 0.009 | Lingual gyrus | Visual |
| 171 | -26 | -90 | 3 | 0.016 | 0.01 | Middle occipital gyrus | Visual |
| 175 | 48 | 25 | 27 | 0.017 | 0.007 | Inferior frontal gyrus, triangular part | Frontoparietal |
| 196 | 40 | 18 | 40 | 0.0009 | 0.009 | Middle frontal gyrus | Frontoparietal |
| 223 | -2 | -13 | 12 | 0.044 | 0.005 | Thalamus | Subcortical |
| 228 | -15 | 4 | 8 | 0.011 | 0.007 | Lenticular nucleus | Subcortical |
|  | | | | | | | |
| **TDC - Medicated** | | | | | | | |
| **ROI** | **MNI Space** | | | **P value (raw)** | **Difference** | **Name** | **Network** |
|  | **X** | **Y** | **Z** |  |  |  |  |
| 15 | 0 | -15 | 47 | 0.003 | 0.008 | Middle cingulate | Sensorimotor hand |
| 26 | 50 | -20 | 42 | 0.02 | 0.006 | Postcentral gyrus | Sensorimotor hand |
| 61 | 32 | -26 | 13 | 0.04 | 0.005 | Heschl gyrus | Auditory |
| 118 | -58 | -30 | -4 | 0.03 | 0.005 | Middle temporal gyrus | Default mode |
| 119 | 65 | -31 | -9 | 0.04 | 0.005 | Middle temporal gyrus | Default mode |
| 120 | -68 | -41 | -5 | 0.005 | 0.007 | Middle temporal gyrus | Default mode |
| 200 | 43 | 49 | -2 | 0.02 | -0.005 | Inferior frontal gyrus, orbital part | Frontoparietal |

**Supplementary Figures**

**Figure S1.** Brain dynamic features. Statistical comparisons of the modularity (a), number of modules (b), average module size (c) and module stationarity (d) between TDC and ADHD subjects. Statistical significance levels: $*p<0.05$, $**p<0.01$, $***p<0.001$.

**Figure S2.** (a) Spatial distribution of the regions with reduced neural flexibility in ADHD when compared to TDC. (b) Number of brain regions with reduced neural flexibility in ADHD when compared to TDC summarized by predefined brain canonical networks (raw $p<0.05$). The total numbers of each predefined canonical network were shown with white bars. The percentages of regions in each network with altered neural flexibility were labeled.

**Figure S3.** (a) Inter-regional correlation of 11 core regions. (b) Inter-regional probability of core regions assigned to same modules across sliding windows.

**Figure S4.** Medication influence at network level. (a) Comparisons of the neural flexibility among different functional networks between medicated ADHD subjects and medication native ADHD subjects. (b) Comparisons of the neural flexibility among different functional networks between medicated ADHD subjects and TDC.

**Figure S5.** Sex difference. A boxplot shows significantly decreased whole brain neural flexibility in female subjects when compared to male subjects using entire dataset ($p=0.0008$). Statistical significance levels: $*p<0.05$, $**p<0.01$, $***p<0.001$.


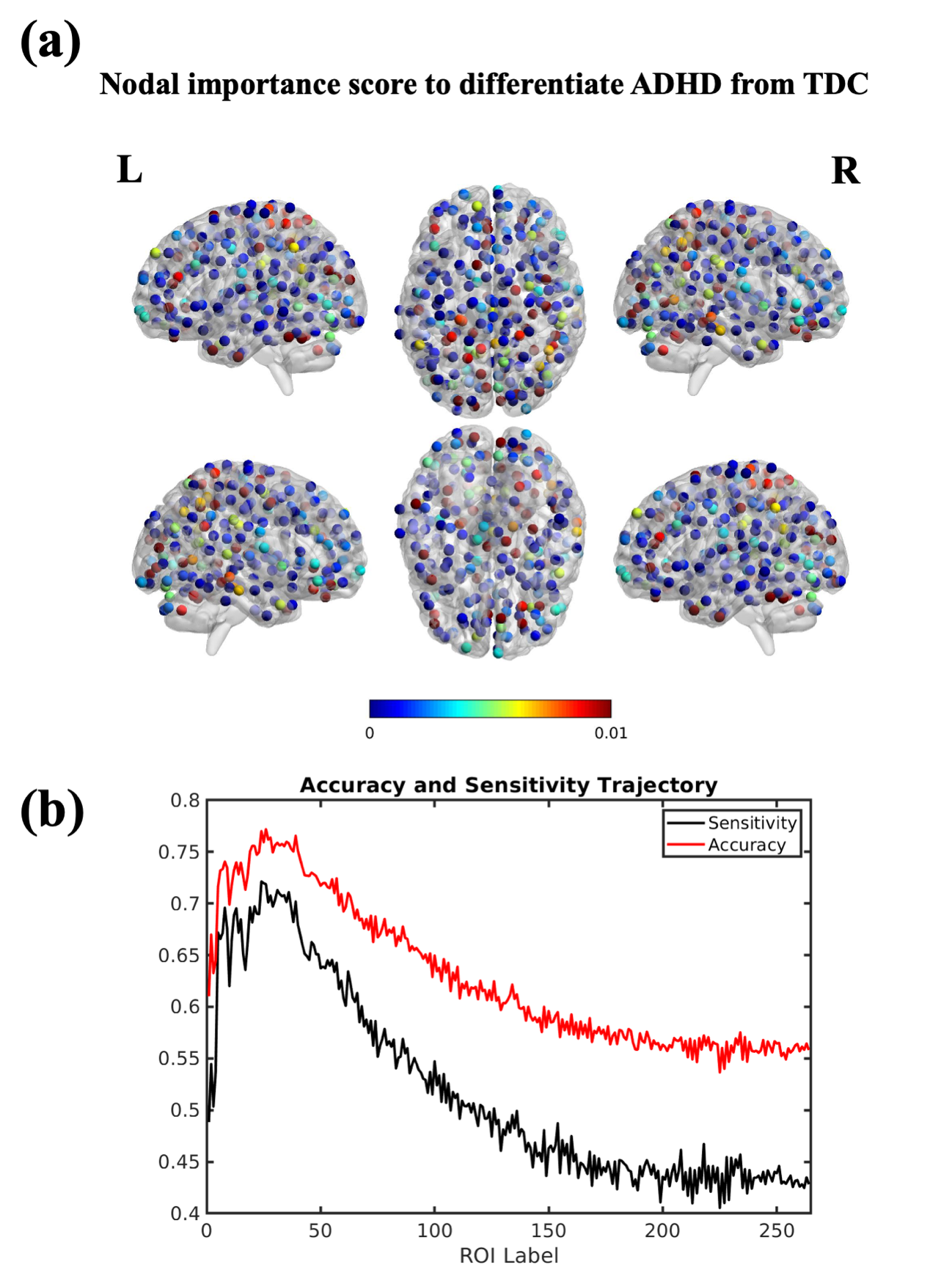


**Figure S6.** Detecting core regions of classification model. (a) The spatial distribution of regional importance scores to differentiate ADHD from TDC. (b) The accuracy and sensitivity trajectory of identifying ADHD subjects using top N most important regions.


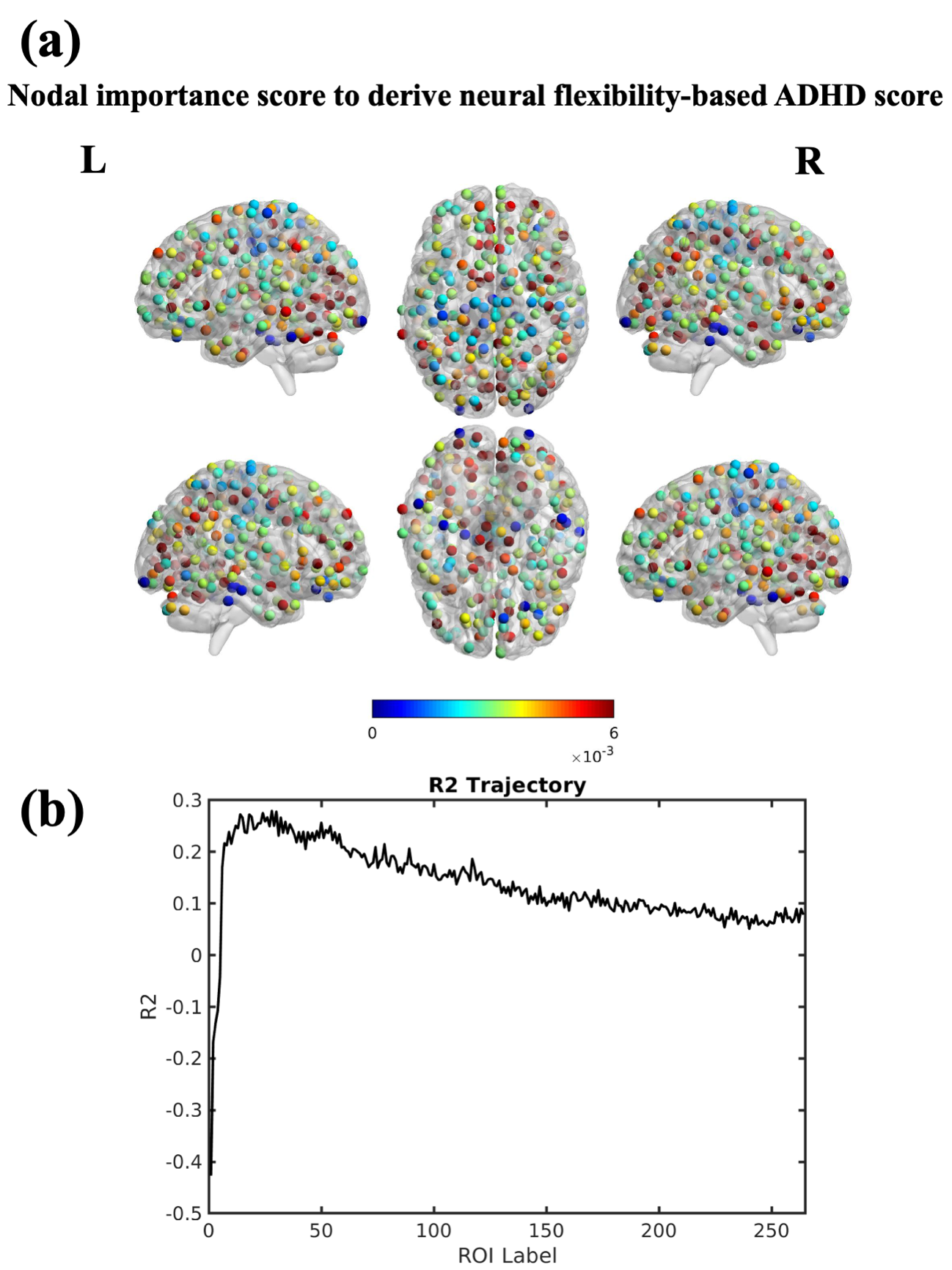


**Figure S7.** Detecting core regions of regression model. (a) The spatial distribution of regional importance score to derive neural flexibility-based ADHD score. (b) The R^2^ score trajectory of the prediction of ADHD index by using top N most important regions.

**Figure S8.** (a) Comparison of the neural flexibility between TDC and ADHD using pediatric-specific templates ($p=0.0008$). (b) Comparison of neural flexibility between using adult MNI and pediatric specific templates ($p=0.76$). (c) Spatial similarity of the distribution of neural flexibility between using adult template and using pediatric templates. Specifically, spatial similarity was calculated as the Pearson’s correlation of neural flexibility across ROIs using pediatric templates and adult MNI templates. Statistical significance levels: $*p<0.05$, $**p<0.01$, $***p<0.001$.

**Figure S9.** (a) Comparisons of the neural flexibility between TDC and ADHD using a fixed network density of 10%, showing that the ADHD group exhibited a significantly lower neural flexibility than that of the TCD group ($p=0.04$). (b) Spatial similarity of the distributions of neural flexibility between using a fixed network density threshold and a statistical significance threshold, respectively. Statistical significance levels: $*p<0.05$, $**p<0.01$, $***p<0.001$.

**Figure S10.** Effects of the resolution and coupling parameters. Comparison of the neural flexibility between TDC and ADHD using different choices of γ and ω. Consistent findings were observed for $0.75\leq\gamma\leq2$ and $0.5\leq\omega\leq2$.

**Figure S11.** Effects of the resolution and coupling parameters. Spatial similarity of the regional distribution of neural flexibility using different choices of γ and ω.

**Figure S12.** (a) Comparison of neural flexibility between TDC and ADHD without spatial smoothing ($p=0.015$). (b) Spatial similarity of the distribution of neural flexibility using 6mm spatial smoothing and without spatial smoothing, respectively. Statistical significance levels: $*p<0.05$, $**p<0.01$, $***p<0.001$.

**Figure S13.** The effects of window length on neural flexibility. Statistical comparisons of whole brain neural flexibility using a window length of (a) 40s ( $p=0.0001$), (b) 60s ($p=0.0005$) and (c) 80s ( $p=0.012$) between TDC and ADHD subjects, respectively. (d) Spatial similarity of the distribution of neural flexibility (60s vs 40s). (e) Spatial similarity of the distribution of neural flexibility (60s vs 80s). Statistical significance levels: $*p<0.05$, $**p<0.01$, $***p<0.001$.

**Supplementary References**

1. Shaffer D, Fisher P, Lucas C. The Diagnostic Interview Schedule for Children (DISC). *Comprehensive handbook of psychological assessment, Vol. 2: Personality assessment.* John Wiley & Sons, Inc.: Hoboken, NJ, US, 2004, pp 256-270.

2. Kaufman J, Birmaher B, Brent D, Rao U, Flynn C, Moreci P *et al.* Schedule for Affective Disorders and Schizophrenia for School-Age Children-Present and Lifetime Version (K-SADS-PL): initial reliability and validity data. *J Am Acad Child Adolesc Psychiatry* 1997; **36**(7)**:** 980-988.

3. Gurley JR. Conners’ Parent Rating Scales – Revised. In: Goldstein S, Naglieri JA (eds). *Encyclopedia of Child Behavior and Development*. Springer US: Boston, MA, 2011, pp 404-405.

4. DuPaul GJ, Power TJ, Anastopoulos AD, Reid R. *ADHD Rating Scale—IV: Checklists, norms, and clinical interpretation*. Guilford Press: New York, NY, US, 1998, viii, 79-viii, 79pp.

5. Li D, Jin Y, Vandenberg SG, Zhu YM, Tang CH. Report on Shanghai norms for the Chinese translation of the Wechsler Intelligence Scale for Children-Revised. *Psychol Rep* 1990; **67**(2)**:** 531-541.

6. Wechsler D. *WASI-II: Wechsler abbreviated scale of intelligence*. PsychCorp2011.

7. Patel AX, Kundu P, Rubinov M, Jones PS, Vertes PE, Ersche KD *et al.* A wavelet method for modeling and despiking motion artifacts from resting-state fMRI time series. *NeuroImage* 2014; **95:** 287-304.

8. Patel AX, Bullmore ET. A wavelet-based estimator of the degrees of freedom in denoised fMRI time series for probabilistic testing of functional connectivity and brain graphs. *NeuroImage* 2016; **142:** 14-26.

9. Avants BB, Tustison NJ, Song G, Cook PA, Klein A, Gee JC. A reproducible evaluation of ANTs similarity metric performance in brain image registration. *NeuroImage* 2011; **54**(3)**:** 2033-2044.

10. Power Jonathan D, Cohen Alexander L, Nelson Steven M, Wig Gagan S, Barnes Kelly A, Church Jessica A *et al.* Functional Network Organization of the Human Brain. *Neuron* 2011; **72**(4)**:** 665-678.

11. Mucha PJ, Richardson T, Macon K, Porter MA, Onnela JP. Community structure in time-dependent, multiscale, and multiplex networks. *Science* 2010; **328**(5980)**:** 876-878.

12. Lucas G. S. Jeub MB, Inderjit S. Jutla, and Peter J. Mucha. “*A generalized Louvain method for community detection implemented in MATLAB*”. [*http://netwikiamathuncedu/GenLouvain*](http://netwikiamathuncedu/GenLouvain)*,* [*https://githubcom/GenLouvain*](https://githubcom/GenLouvain) *(2011-2019)*.

13. Yin W, Li T, Hung SC, Zhang H, Wang L, Shen D *et al.* The emergence of a functionally flexible brain during early infancy. *Proc Natl Acad Sci U S A* 2020.

14. Bassett DS, Wymbs NF, Porter MA, Mucha PJ, Carlson JM, Grafton ST. Dynamic reconfiguration of human brain networks during learning. *Proceedings of the National Academy of Sciences of the United States of America* 2011; **108**(18)**:** 7641-7646.

15. Palla G, Barabasi AL, Vicsek T. Quantifying social group evolution. *Nature* 2007; **446**(7136)**:** 664-667.

16. Chen T, Guestrin C. XGBoost: A Scalable Tree Boosting System. *Proceedings of the 22nd ACM SIGKDD International Conference on Knowledge Discovery and Data Mining*. Association for Computing Machinery: San Francisco, California, USA, 2016, pp 785–794.

17. Le NQK, Do DT, Chiu FY, Yapp EKY, Yeh HY, Chen CY. XGBoost Improves Classification of MGMT Promoter Methylation Status in IDH1 Wildtype Glioblastoma. *J Pers Med* 2020; **10**(3).

18. Inoue T, Ichikawa D, Ueno T, Cheong M, Inoue T, Whetstone WD *et al.* XGBoost, a Machine Learning Method, Predicts Neurological Recovery in Patients with Cervical Spinal Cord Injury. *Neurotrauma Reports* 2020; **1**(1)**:** 8-16.

19. Sharma A, Verbeke WJMI. Improving Diagnosis of Depression With XGBOOST Machine Learning Model and a Large Biomarkers Dutch Dataset (n = 11,081). *Frontiers in Big Data* 2020; **3**(15).

20. Torlay L, Perrone-Bertolotti M, Thomas E, Baciu M. Machine learning-XGBoost analysis of language networks to classify patients with epilepsy. *Brain Inform* 2017; **4**(3)**:** 159-169.

21. Bianchi J, de Oliveira Ruellas AC, Gonçalves JR, Paniagua B, Prieto JC, Styner M *et al.* Osteoarthritis of the Temporomandibular Joint can be diagnosed earlier using biomarkers and machine learning. *Scientific Reports* 2020; **10**(1)**:** 8012.

22. Polikar R. Ensemble based systems in decision making. *IEEE Circuits and Systems Magazine* 2006; **6**(3)**:** 21-45.

23. Castellanos FX, Aoki Y. Intrinsic Functional Connectivity in Attention-Deficit/Hyperactivity Disorder: A Science in Development. *Biol Psychiatry Cogn Neurosci Neuroimaging* 2016; **1**(3)**:** 253-261.

24. Rolls ET, Cheng W, Feng J. Brain dynamics: the temporal variability of connectivity, and differences in schizophrenia and ADHD. *Transl Psychiatry* 2021; **11**(1)**:** 70.

25. Zhao T, Liao X, Fonov VS, Wang Q, Men W, Wang Y *et al.* Unbiased age-specific structural brain atlases for Chinese pediatric population. *NeuroImage* 2019; **189:** 55-70.

26. Fonov V, Evans AC, Botteron K, Almli CR, McKinstry RC, Collins DL *et al.* Unbiased average age-appropriate atlases for pediatric studies. *NeuroImage* 2011; **54**(1)**:** 313-327.

27. Bassett DS, Porter MA, Wymbs NF, Grafton ST, Carlson JM, Mucha PJ. Robust detection of dynamic community structure in networks. *Chaos* 2013; **23**(1)**:** 013142.

28. Bassett DS, Yang M, Wymbs NF, Grafton ST. Learning-induced autonomy of sensorimotor systems. *Nat Neurosci* 2015; **18**(5)**:** 744-751.

29. Gao W, Gilmore JH, Giovanello KS, Smith JK, Shen D, Zhu H *et al.* Temporal and Spatial Evolution of Brain Network Topology during the First Two Years of Life. *PloS one* 2011; **6**(9)**:** e25278.

30. Yin W, Chen M-H, Hung S-C, Baluyot KR, Li T, Lin W. Brain functional development separates into three distinct time periods in the first two years of life. *NeuroImage* 2019; **189:** 715-726.

31. Wang J, Dong Q, Niu H. The minimum resting-state fNIRS imaging duration for accurate and stable mapping of brain connectivity network in children. *Sci Rep* 2017; **7**(1)**:** 6461.

32. Weir WH, Emmons S, Gibson R, Taylor D, Mucha PJ. Post-Processing Partitions to Identify Domains of Modularity Optimization. *Algorithms* 2017; **10**(3)**:** 93.

33. Blondel VD, Guillaume J-L, Lambiotte R, Lefebvre E. Fast unfolding of communities in large networks. *Journal of Statistical Mechanics: Theory and Experiment* 2008; **2008**(10)**:** P10008.

34. Yin W, Li T, Hung S-C, Zhang H, Wang L, Shen D *et al.* The emergence of a functionally flexible brain during early infancy. *Proceedings of the National Academy of Sciences* 2020; **117**(38)**:** 23904-23913.

35. Braun U, Schafer A, Walter H, Erk S, Romanczuk-Seiferth N, Haddad L *et al.* Dynamic reconfiguration of frontal brain networks during executive cognition in humans. *Proceedings of the National Academy of Sciences of the United States of America* 2015; **112**(37)**:** 11678-11683.

36. Braun U, Schafer A, Bassett DS, Rausch F, Schweiger JI, Bilek E *et al.* Dynamic brain network reconfiguration as a potential schizophrenia genetic risk mechanism modulated by NMDA receptor function. *Proceedings of the National Academy of Sciences of the United States of America* 2016; **113**(44)**:** 12568-12573.

37. Lei T, Liao X, Chen X, Zhao T, Xu Y, Xia M *et al.* Progressive Stabilization of Brain Network Dynamics during Childhood and Adolescence. *Cerebral cortex* 2021.

38. Power JD, Barnes KA, Snyder AZ, Schlaggar BL, Petersen SE. Spurious but systematic correlations in functional connectivity MRI networks arise from subject motion. *NeuroImage* 2012; **59**(3)**:** 2142-2154.

39. Leonardi N, Van De Ville D. On spurious and real fluctuations of dynamic functional connectivity during rest. *NeuroImage* 2015; **104:** 430-436.

40. Zalesky A, Breakspear M. Towards a statistical test for functional connectivity dynamics. *NeuroImage* 2015; **114:** 466-470.

41. Hutchison RM, Womelsdorf T, Allen EA, Bandettini PA, Calhoun VD, Corbetta M *et al.* Dynamic functional connectivity: promise, issues, and interpretations. *NeuroImage* 2013; **80:** 360-378.

42. Hutchison RM, Womelsdorf T, Gati JS, Everling S, Menon RS. Resting-state networks show dynamic functional connectivity in awake humans and anesthetized macaques. *Human brain mapping* 2013; **34**(9)**:** 2154-2177.

43. Shirer WR, Ryali S, Rykhlevskaia E, Menon V, Greicius MD. Decoding Subject-Driven Cognitive States with Whole-Brain Connectivity Patterns. *Cerebral cortex* 2011; **22**(1)**:** 158-165.

44. Cohen JR. The behavioral and cognitive relevance of time-varying, dynamic changes in functional connectivity. *NeuroImage* 2018; **180**(Pt B)**:** 515-525.
